# A method to prevail false positive responses due to excess cations and viscous nature of Radiopharmaceuticals in Limulus Amebocyte Lysate Gel Clot test

**DOI:** 10.22038/AOJNMB.2021.59607.1416

**Published:** 2022

**Authors:** Arpit Mitra, Sangita Lad, Sudeep Sahu, Savita Kulkarni

**Affiliations:** 1 Medical Cyclotron Facility, Radiation Medicine Centre, Board of Radiation and Isotope Technology, Parel, Mumbai, India; 2 Radiation Medicine Centre, Bhabha Atomic Research Centre, Parel, Mumbai, India; 3 Homi Bhabha National Institute, Mumbai, India

**Keywords:** Limulus Amebocyte Lysate Gel-Clot test, Lutetium-177-DOTATATE, Iodine-131-Lipiodol, Transglutaminase

## Abstract

**Objective(s):**

Bacterial endotoxin test (BET) for detection and quantification of endotoxin in radiopharmaceuticals (RPs), used for therapy or diagnosis, is prerequisite to administration in patients. Out of the two established methods used for this purpose (Kinetic Chromogenic Assay: KCM and Gel Clot Bacterial Endotoxin Test: GC-BET), GC-BET is recommended by pharmacopeias to evaluate the interferences exhibited during the assay due to presence of various ingredients in samples. In the present study, the influence of excess of cations in [^177^Lu]Lu-DOTATATE, used for Peptide Receptor Radionuclide Therapy (PRRT), were studied and a protocol to negate the enhancement observed was developed. Additionally, a protocol for carrying out GC-BET for extremely viscous [^131^I]I-Lipiodol was standardized.

**Methods:**

GC-BET was performed for [^177^Lu]Lu-DOTATATE and [^131^I]I-Lipiodol at maximum valid dilution (MVD), using LRW as a diluent. To negate the false positivity observed in case of [^177^Lu]Lu-DOTATATE, various concentrations of calcium chloride (CaCl_2_) were added and evaluated for the reversal of the interference observed initially. To prevail the difficulty in performing GC-BET for [^131^I]I-Lipiodol various modification in the protocols like orbital vortexing at different rpm and time intervals were performed. KCM assays were also performed for studied RPs at MVD.

**Results:**

It was observed that at MVD, [^177^Lu]Lu-DOTATATE exhibited false positivity in GC-BET. However, all the individual reagents used in labeling of [^177^Lu]Lu-DOTATATE did not show any false positivity. Finally, performing the assay with an addition of 2mM CaCl_2_ (final concentration) nullified the false positivity. Further, intricacy in performing GC-BET for [^131^I]I-Lipiodol due to its viscosity was resolved by orbital vortexing at 3000 rpm for 5 minutes.

**Conclusions:**

Our study proved that false positivity was observed in GC-BET for [^177^Lu]Lu-DOTATATE due to the presence excess M^3+^ ions. Further, our study is the first of its kind which demonstrated methods for negating these false positive results by using modified protocol and hypothesizing the reason behind the enhancement. Additionally, ours is the first study which proved that a simple step of vortexing the viscous RPs like [^131^I]I-Lipiodol can resolved the problems encountered during performing GC-BET due to viscosity of RPs.

## Introduction

 The use of radio-labeled peptides like ([^177^Lu]Lu-DOTATATE) for PRRT has enhanced effectiveness in cancer treatment because of selective delivery of radioisotope to tumor sites which further reduces toxic side effects to healthy tissues. Additionally, target specific radio-labeled fatty acid (RFA) like [^131^I]I-Lipiodol and [^177^Lu]Lu-DOTATATE, a radio-lanthanide labeled RP (RL-RP), has been extensively used for treatment of liver metastases and have obtained an approval from European Medicines Agency (EMA) and United States Food and Drug Administration (USFDA) in recent years (1,2). The success of RPs lies on the selection of suitable peptides, fatty acids, chelating cores and their ability to form stable conjugate and further to achieve efficient radio-labeling with various radioisotopes like Lutetium-177, Yttrium-90, Rhenium-188 and Technetium-99m.

 Most of these target specific therapeutic RPs are approved by USFDA and the EMA for treatment of various cancers in humans and these therapeutic RPs are considered to be in generic form. Hence, it is mandatory to test these radiolabeled formulations for apyro-genicity prior to administration in patients, to ensure that endotoxin levels are within permissible limits.

 For RL-RPs, Endotoxin Limit (EL) is 175 EU/V (3, 4) whereas for intra-arterial infused RFA, EL is 17.8 EU/mL (5, 6). The standard method for assessing the bacterial endotoxin levels in any of these studied RPs and their cold kits is by rabbit pyrogen test as described in United State Pharmacopoeia (USP) chapter 151. However, common limitations with this test are; its labor intensive nature, potential radioactive dose to analyst and problems during administering radioactivity to animals.

 GC-BET is the most economical and technically uncomplicated method available for evaluation of bacterial endotoxin in radiopharmaceuticals. However, it is very sensitive towards concentration of divalent calcium ions and the change in concentration during assay, contributed by the samples, lead to interference either in the form inhibition or enhancement. Radiometals like ^177^Lu, ^90^Y, ^188^Re, ^225^Ac etc are used for preparation of rdaiopharmaceuticals for therapeutic purpose and these radiometals undergo radiometallation or radiochelation reaction with suitable cyclic / acyclic / bifunc-tional chelators to which target specific peptides / enzyme inhibitors / monoclonal anti-bodies are attached. These final radiopharma-ceutical preparations contain various mono-valent / divalent / trivalent non-radioactive cations which do not interfere with physio-logical uptake. However, these cations, crucially get involved during enzymatic reaction of GC-BET and compete with Ca^2+^ ions, thereby increasing (enhancement) or decreasing (inhibition) the sensitivity of assay. The enhancement observed in the assay results in false positivity, while inhibition leads to false negativity.

 The present study was undertaken to evaluate influence of non-radioactive or radioactive cations (present in radiopharmaceutical formulations) on transglutaminase mediated coagulation reaction during GC-BET. Further, protocols were developed to overcome the false positivity observed in case of [^177^Lu]Lu-DOTATATE and [^131^I]I-Lipiodol, due to the influence of cations and viscous nature of radiopharmaceuticals respectively in Limulus Amebocyte Lysate Gel Clot test. The study also demonstrated the feasibility for carrying out GC-BET assay by a modified methodology for [^131^I]I-Lipiodol, a highly viscous radiopharma-ceutical infused through intra-arterial route.

## Materials

 LAL Reagent- Lysate (Sensitivity [λ]: 0.125 and 0.03 EU/mL), Control Standard Endotoxin CSE (Stock Solution Concentration is 20 and 40 EU/mL), LAL Reagent Water (LRW, Endotoxin level < 0.001 EU/mL), Portable Test System Kinetic Reader (PTS), PTS test cartridges (sensitivity[λ]: 5-0.05 EU/mL) were procured from Charles River Laboratories, USA. Endotoxin stock solution was prepared in accordance with the information stated in the certificate of analysis (COA). Sterile and endotoxin free 60 mM solution of calcium chloride was prepared in-house. [^131^I]I-Lipiodol was received from Radiopharma-ceutical Division of Bhabha Atomic Research Centre (BARC), India. [^177^Lu]LuCl_3_ used for radiolabeling of PRRT agents was obtained from BARC Mumbai, India. 1, 4, 7, 10-tetraazacyclo-dodecane-N, N', N'', N'''-tetraaceticacid-Tyr3-octreotate (DOTATATE) was procured from Auspep Pty, Australia and was reconstituted with ultrapure water from Aldrich, USA (concentration: 1 mg/mL, screened in advance for endotoxin) and filtered through 0.22 µm sterile filter (Millipore, Germany). Sterile endotoxin free ammonium acetate buffer (0.1 M, pH=4) was prepared by dissolving ammonium acetate, gentisic acid and sodium hydroxide (Aldrich, USA) in ultrapure water and filtering through 0.22 µm sterile filter. Radio- labeling of DOTATATE with [^177^Lu]LuCl_3_ was carried out as per reported procedures (7).

## Methods

 In BET assay, it is mandatory to calculate the Maximum Valid Dilution (MVD) which is the maximum allowable dilution of a sample at which the endotoxin limit can be determined and interfering test conditions can be avoided. MVD = EL/λ (Where EL is Endotoxin Limit of RPs and λ is Sensitivity in EU/mL of LAL reagent used). Considering the EL of studied RPs, MVD is calculated and sensitivity at which assay is to be performed is described in Table 1. Detailed protocol for GC-BET is shown in Table 2, and the assays were performed for all the RPs at MVD.

**Table 1:** EL, MVD and Sensitivity used in GC-BET assay for various RPs and APIs

**Products**	**Endotoxin Limit**	**MVD**	**Sensitivity of Lysate**
[^177^Lu]Lu-DOTATATE ([^177^Lu]Lu^3+^: 0.226 µmol, RCP: 90 to <95%)	<4	30	0.125
[^177^Lu]Lu-DOTATATE ([^177^Lu]Lu^3+^: 0.226 µmol, RCP: ≥ 95 to 99%)	<4	30	0.125
[^131^I]I-Lipiodol	<12	400	0.03
[^177^Lu]LuCl3 ([^177^Lu]Lu^3+^: 0.226 µmol)			
DOTATATE acetate (0.565 µmol)	<4	30	0.125
Ammonium acetate buffer (0.1 M)	<4	30	0.125
Gentisic acid (0.26 M)	<4	30	0.125

**Table 2:** The detailed protocol for the GC-BET performed

**Test Serial No**	**Assay Tube (In Duplicate)**	**LRW ** **(µL)**	**CSE (4λ)** ** (µL)**	Test Sample* ‡ §? (µL)	**LAL Reagent (µL)**
1	Negative Water Control	100	…	…	100
2	Positive Water Control 2λ	50	50	…	100
3	Negative Product Control†	50	…	50	100
4	Positive Product Control 2λ†	…	50	50	100

Test 3 and 4 were also performed initially for stock solution of CaCl_2_ solution, saline (0.9% sodium chloride) and also for ultrapure water to ensure absence of endotoxin in stock solutions.

*: Done at MVD for [^131^I]I-Lipiodol and [^177^Lu]Lu-DOTATATE.

†: These tubes were set up for [^131^I]I-Lipiodol.

‡: First dilution tubes of test sample (MVD/2) were vortexed at 3000 rpm for 8 minutes for [^131^I]I-Lipiodol

§: 100 µl of excess CaCl_2 _(60 mM) was incorporated in first dilution tube (MVD/2) of test sample using LRW as diluents for [^177^Lu]Lu-DOTATATE so the 2 mM concentration of CaCl_2_ was maintained in the final assay. Assay was also performed in same way with excess of Mg^2+^.

?: For [^177^Lu]LuCl3, ammonium acetate buffer and gentisic acid, pH of the test samples were adjusted using 0.25 M tris base at first dilution. pH adjustment prior to assay procedure, were not required for DOTATATE acetate.

 The tests, Negative Product Controls (NPC) and Positive Product Controls (PPC) were incubated at 37^o^C for 60 minutes in a dry heating block. To demonstrate reproducibility of the results 3 batches of each of RPs were tested. To demonstrate that the usage of CaCl_2_ does not interfere in the gel-clot formation and also does not reduce or enhance the sensitivity of the test, we have carried out assays with positive water control at different standard endotoxin concentrations with and without calcium chloride. The assay has also been done with negative water control with and without calcium chloride. The assays were repeated 10 times to study reproducibility. For all the studied RPs and active product ingredients (APIs), KCM assays using PTS were performed at the same MVD at which GC-BET were performed. Detailed protocol for the procedure is shown in Table 3.

**Table 3:** The detailed protocol for the KCM BET

Products	MVD	Test Samples (µL)	LRW (µL)
[^177^Lu]Lu-DOTATATE ([^177^Lu]Lu^3+^: 0.226 µmol, RCP: 90 to <95%)	30	20	580
[^177^Lu]Lu-DOTATATE ([^177^Lu]Lu^3+^: 0.226 µmol, RCP: ≥95 to 99%)	30	20	580
[^177^Lu]LuCl_3_^*^ ([^177^Lu]Lu^3+^: 0.226 µmol)	30	20	580
Ammonium acetate buffer (0.1 M)^*^	30	20	580
DOTATATE acetate (0.565 µmol)	30	20	580

*: pH of test Samples was adjusted with 0.25 M tris base prior KCM-BET

 In a typical radiolabelling procedures, carrier added [^177^Lu]LuCl_3_ (445 µL, specific activity: 0.74 GBq/µg) solution (29.6 GBq of [^177^Lu]Lu^3+^, 40 µg, 0.226 µmol Lu) in 0.01M HCl was added to a sterile glass vial containing a mixture of DOTATATE solution (811 µL of 1 mg/mL solution in ultrapure grade water, 0.565 µmol, 2.5 equivalent of Lu) and gentisic acid dissolved in CH3COONH4 buffer (0.1 M, 3.6 mL). 

 For GC-BET and KCM assay carried out for [^177^Lu]LuCl_3_ and DOTATATE, the dilution were carried out in such a way, that the concen-tration of [^177^Lu]LuCl_3_ and DOTATATE test samples were maintained at 0.226 µmol and 0.565 µmol respectively.

## Results

 GC-BET results for [^131^I]I-Lipiodol at 400 MVD are depicted in Table 4. Even at MVD as high as 400, it is unfeasible to perform successful GC-BET for [^131^I]I-Lipiodol without any modification, due to high viscosity of lipiodol and thick precipitation were observed in NPC test sample. However, this precipitation in NPC can be overcome by carrying out orbital vortexing at 3000 rpm for 5-8 minutes, at first dilution of the test samples. This short orbital vortexing step allows the dispersion of the viscous layer formed during dilution of samples.

**Table 4:** Results of [^131^I]I-Lipiodol at MVD in GC-BET

**Product Analyzed**	**Maximum Valid Dilution (MVD) i.e 1:400**								
	** NPC**				** PPC**				
	**No Vortexing**		**Votexing at 3000 rpm**		**No Vortexing**			**Votexing at 3000 rpm**	
[^131^I]I-Lipiodol	++	++	--	--	++	++	++		++

NPC: Negative Product Control, PPC: Positive Product Control, rpm: rotation per minute

++: Firm Gel Clot, --: No Gel Clot

 On performing GC-BET for [^177^Lu]Lu-DOTATATE, whose Radiochemical Purity (RCP) was between 90 to <95%; the NPC test samples illustrated firm clot at 30 MVD (Table 5). The firm clot in NPC indicated false positive results due to increased concentration of [^177^Lu]Lu^3^+ in the final product. These false positive results were reversed on addition of 2 mM Ca^2+^ ions (CaCl_2_ form) in test samples (final assay). Results from Table 5, also show that the addition of same concentration of Mg^2+^ ions (MgCl_2_ form) could not circumvent the false positive results in NPC test samples at 30 MVD. Hence, it can be concluded that the concentration of Ca^2+^ ions plays a predominant role in transglutaminase mediated coagulation reaction which is the prime reaction in gel clot formation.

 The GC-BET assay for [^177^Lu]Lu-DOTATATE with higher RCP (≥95 to 99%) did not exhibit any false positive results in NPC test samples at 30 MVD. The observed results (Table 5) strongly support the fact that the concentration of Ca^2+^ ions plays a very critical role and it gets affected by increased concentration of monovalent or trivalent cations in test samples. Further, the GC-BET results (Table 5) for individual APIs used in radiolabelling like [^177^Lu]LuCl_3_ (0.226 µmol), DOTATATE (0.565 µmol), ammonium acetate buffer (0.1 M) and gentisic acid (0.26 M) did not exhibit any false positivity (firm clot formation) in NPC test samples at 30 MVD. The exhibited result indicate that the used APIs are not responsible for exhibiting false positive results in [^177^Lu]Lu-DOTATATE test samples (RCP: 90 to <95%).

**Table 5:** Results observed for GC-BET assay for different RL-RP and and APIs at MVD

	**At 30 Maximum Valid Dilution (MVD)**					
	**NPC**			**PPC**		
	**withoutCa** ^2+^ **/Mg** ^2+^	**with #Ca** ^2+^	**with $Mg** ^2+^	**without Ca** ^2+^ **/Mg** ^2+^	**with #Ca** ^2+^	**with $Mg** ^2+^
[^177^Lu]Lu-DOTATATE ([^177^Lu]Lu^3+^:0.226 µmol, RCP: 90 to <95%)	--	--	--	++	++	++
[^177^Lu]Lu-DOTATATE ([^177^Lu]Lu^3+^: 0.226 µmol, RCP: ≥95 to 99%)	++	--	++	++	++	++
[^177^Lu]LuCl_3_ ([^177^Lu]Lu^3+^:0.226 µmol)	--	--	--	++	++	++
DOTATATE acetate (0.565 µmol)	--	--	--	++	++	++
Ammonium acetate buffer (0.1 M)						
Gentisic acid (0.26 M)	--	--	--	++	++	++

#Ca^2+^: 2 mM of Ca^2+^ (CaCl_2_ form) in the test sample at final assay.

$Mg^2+^: 2 mM of Mg^2+ ^(MgCl_2_ form) in the test sample at final assay.

NPC: Negative Product Control, PPC: Positive Product Control

++: Firm Gel Clot, --: No Gel Clot

 The percentage for recovery of positive product control (RPPC) of [^177^Lu]Lu-DOTATATE (RCP: 90 to <95% ), [^177^Lu]Lu-DOTATATE (RCP: ≥95 to 99%), [^177^Lu]LuCl_3 _(0.226 µmol), DOTATATE (0.565 µmol) and ammonium acetate buffer (0.1 M) were 125%, 118%, 96%, 90% and 109% respectively at 30 MVD (Table 6). RPPC for all the test samples were between 50-200%, indicating no interferences in KCM assay performed by PTS. RPPC of [^177^Lu]Lu-DOTATATE (RCP: 90 to <95% ) test samples KCM assay was 125% even without addition of 2 mM Ca^2+^ ions (CaCl_2_ form) in test samples at 30 MVD.

**Table 6:** Results observed for KCM BET assay for different RL-RP and and APIs at MVD

**Products Analyzed**	**RPPC (%)**	EL *(EU / mL)	**MVD**
[^177^Lu]Lu-DOTATATE ([^177^Lu]Lu^3+^: 0.226 µmol, RCP: 90 to <95%)	125	<1.5	30
[^177^Lu]Lu-DOTATATE ([^177^Lu]Lu^3+^: 0.226 µmol, RCP: ≥95 to 99%)	118	<1.5	30
[^177^Lu]LuCl_3_^*^ ([^177^Lu]Lu^3+^: 0.226 µmol)	96	<1.5	30
DOTATATE acetate (0.565 µmol)	109	<1.5	30
Ammonium acetate buffer (0.1 M)^*^	90	<1.5	30

*: 5 – 0.05 EU/ml is the sensitivity of KCM – BET

Table 7 shows the result obtained for the GC-BET carried out at different concentrations of standard endotoxin from 4λ to λ/4 (λ: sensitivity of lysate used i.e 0.125 EU/mL and 0.03 EU/mL) and it can be seen that the presence of CaCl_2_ does not interfere in the assay. Further, the experiments gave consistent results over 10 replications indicating the validity and reproducibility of the procedure of reversing the enhancement of gel clot formation after addition of excess CaCl_2_.

**Table 7:** Results observed for GC BET assay performed at different standard endotoxin concentrations in the presence and absence of calcium chloride

**Experiment** **No.**		**(4λ)**			**(2λ)**			**(λ)**			**(λ/2)**			**(λ/4)**		
		**T1**	**T2**	**T3**	**T1**	**T2**	**T3**	**T1**	**T2**	**T3**	**T1**	**T2**	**T3**	**T1**	**T2**	**T3**
**1**	**A**	++	++	++	++	++	++	++	++	++	+	+	+	--	--	--
	**B**	++	++	++	++	++	++	++	++	++	+	+	+	--	--	--
**2**	**A**	++	++	++	++	++	++	++	++	++	+	+	+	--	--	--
	**B**	++	++	++	++	++	++	++	++	++	+	+	+	--	--	--
**3**	**A**	++	++	++	++	++	++	++	++	++	+	+	+	--	--	--
	**B**	++	++	++	++	++	++	++	++	++	+	+	+	--	--	--
**4**	**A**	++	++	++	++	++	++	++	++	++	+	+	+	--	--	--
	**B**	++	++	++	++	++	++	++	++	++	+	+	+	--	--	--
**5**	**A**	++	++	++	++	++	++	++	++	++	+	+	+	--	--	--
	**B**	++	++	++	++	++	++	++	++	++	+	+	+	--	--	--
**6**	**A**	++	++	++	++	++	++	++	++	++	+	+	+	--	--	--
	**B**	++	++	++	++	++	++	++	++	++	+	+	+	--	--	--
**7**	**A**	++	++	++	++	++	++	++	++	++	+	+	+	--	--	--
	**B**	++	++	++	++	++	++	++	++	++	+	+	+	--	--	--
**8**	**A**	++	++	++	++	++	++	++	++	++	+	+	+	--	--	--
	**B**	++	++	++	++	++	++	++	++	++	+	+	+	--	--	--
**9**	**A**	++	++	++	++	++	++	++	++	++	+	+	+	--	--	--
	**B**	++	++	++	++	++	++	++	++	++	+	+	+	--	--	--
**10**	**A**	++	++	++	++	++	++	++	++	++	+	+	+	--	--	--
	**B**	++	++	++	++	++	++	++	++	++	+	+	+	--	--	--

A: with CaCl_2_ , B: without CaCl_2_ , λ: Sensitivity of lysate

T1, T2 and T3 are triplicates for each different standard endotoxin concentration

## Discussion

 The use of peptides, enzyme inhibitor and other molecules, radiolabeled with different radio-isotopes of different half-lives and energies, is ever increasing in treatment as well as diagnosis. In view of the spot preparations of RPs, in house endotoxin evaluation before administration to patients, has become mandatory in hospital radiopharmacy unit of the Nuclear Medicine Departments of hospitals. As mentioned earlier the endotoxin in samples can be estimated using GC-BET or KCM. Coagulation cascade reaction in GC-BET is based on presence of natural substrates i.e. proline rich proteins and 8.6 kDa proteins of limulus transglutaminase present in non-granular hemocytes (L granules) of horse shoe crab8 and also on optimum proteolytic activity of transglutaminase enzyme. Optimum proteolytic activity of transglutaminase is dependent on the balanced free divalent cations concentration9, 10, 11, 12 and overall this concentration in ionic form (Ca^2+^) has to be maintained for optimized transglutaminase activity (13). 

 Transglutaminase binds to three Ca^2+^ ions at three different sites, which means optimum concentration should be 1.0 Ca^2+^/mol in absence of any other metals. The Ca^2+^ ion in site one is constitutively bound with binding affinity of Kd~0.3 µM (ΔH ~ -6.70 kcal/mol) and is required for stabilization of the enzyme (14). Upon proteolysis transglutaminase exothermally acquires two more Ca^2+^ ions at site 2 and 3, which activates the transglutaminase by opening a channel which exposes tryptophan-236 (Trp-236) and tryptophan-327 (Trp-327) residues that controls substrate access to the active site (15). 

 [^177^Lu]Lu-DOTATATE is used for PRRT for patients with neuroendocrine tumors (NET) and in present study, it was prepared at Centralized Radiopharmacy Centre (CPC) with RCP of ≥95 to 99%, using carrier added [^177^Lu]LuCl_3_ as per the standard procedures (7). At CPC when GC-BET was performed at 30 MVD (total production volume is 7-9 ml) along with other QC analysis, the preparation did not show any false positive results. 

 However, when the [^177^Lu]Lu-DOTATATE is stored at -20^o^C for 2-3 days, RCP decreases to 90 to <95% due to formation of free radicals, resulting in increased concentrations of free ionic species of [^177^Lu]Lu^3+^ in the product16. This [^177^Lu]Lu-DOTATATE at RCP of 90 to <95% showed false positivity in GC-BET at 30 MVD. It may be hypothesized that presence of excess free [^177^Lu]Lu^3+^ ions in the test sample may be the main reason of this false positivity. The excess free trivalent cations i.e ^177^Lu^3+^ ions may be comfortably binding to site 2 of transglutaminase due to appropriate ionic radius (6 coordinate: 100 pm and 8 coordinate: 111 pm) and multidentate property similar to free divalent Ca^2+^ ions ionic radius (6 coordinate :114 pm and 8 coordinate: 126 pm) (17). In GC-BET assay, the conversion of coagulogen to coagulin dimer (stiff gel formation) is facilitated by transglutaminase enzyme in presence of three Ca^2+^ ions on heating at 37^o^C for 60 minutes. Avazhi et. al. have reported increased enzymatic activity of transglutaminase when one or two Ca^2+^ are replaced with any of the nonradioactive lanthanide trivalent cations ( Er^3+^, Sm^3+^, Tb^3+^ and Lu^3+^). Similarly increased transglutaminase enzymatic activity due to presence of excess [^177^Lu]Lu^3+^ ions in [^177^Lu]Lu-DOTATATE (RCP: 90 to <95%) does not allow the conversion of coagulogen to coagulin dimer on heating at 37^o^C for 60 minutes, rather gives appearance as thick gel (enhancement or no dimer formation) in negative and positive product control samples (14,15).

 The enhancement was reversed by adding 2 mM Ca^2+^ ions (calcium chloride form) in [^177^Lu]Lu-DOTATATE (RCP: 90 to <95%) BET test sample at 30 MVD. It can be reasoned out that, the increase in concentration of Ca^2+ ^ions leads to binding of excess Ca^2+^ at site 2 and 3 of transglutaminase replacing the presence of excess [^177^Lu]Lu^3+ ^ions and thus facilitating the opening of channel for optimum enzymatic activity. However, enhanced BET result was not observed at RCP 95-99% at same MVD which indicates that free [^177^Lu]Lu^3+^ concentration at this (RCP 95-99%) formulation is not sufficient enough to bind to site 2 and 3 of transglutaminase and does not affect the opening of channel for exposing Trp-236 and Trp-327 residue. 

When BETs were performed at 30 MVD with same concentration (2 mM) of Mg^2+^ ions (magnesium chloride form) instead of Ca^2+^ ions, enhanced BET result could not reversed. This may be because more biologically relevant Mg^2+ ^ions binding at site 2 and 3 of transglutaminase do not expose the channel which controls substrate access to active site14. This result proves the concept of binding three Ca^2+^ ions at 3 different sites is absolutely necessary for optimum transglutaminase enzymatic activity. An enhancement from other sources like raw materials used viz: [^177^Lu]LuCl_3_, DOTATATE acetate (concen-tration: 1 mg/mL), 0.1 M ammonium acetate buffer and 0.26M gentisic acid, was ruled out since results of their GCM- BET did not exhibit any enhancement at 30 MVD using LRW as assay medium. The nonoccurrence of false positive result in GC-BET of [^177^Lu]LuCl_3_ indicates [^177^Lu]Lu^3+ ^ does not exist in free ionic species and rather there exists an ionic bond between [^177^Lu]Lu^3+^ and Cl^-^, since the [^177^Lu]Lu^3+^ is in 0.1M HCl. However, this is not the case with [^177^Lu]Lu-DOTATATE (RCP: 90 to <95%), where [^177^Lu]Lu^3+ ^is present as free ionic species since the [^177^Lu]Lu^3+^ is solvated by water molecules contributed from ammonium acetate buffer. 

 In our earlier published literature (12), we reported that some of the ^99m^Tc- labeled radiopharmaceuticals (^99m^Tc-RPs) and their cold kits (TCKs) exhibited inhibition during GC-BET. The PPC test samples (^99m^Tc-DMSA-III, ^99m^Tc-DMSA-V, ^99m^Tc-EC, ^99m^Tc-DTPA and their respect-tive cold kits) did not form any firm clots and false negative results were observed. These false negative results were reversed by adding excess 300 µM Ca^2+^ ions (CaCl_2_ form) in test samples at MVD. We reported that, the free carboxyl groups (-COOH) present in some of the ^99m^Tc-RPs and their respective TCKs inhibit GC-BET by depleting Ca^2+^ ions concentration, thereby inhibiting transglutaminase activity. However, though DOTATATE acetate, was with presence of free –COOH groups, no false negative results in PPC test samples were observed. This may be because the molar concentration for DOTATATE acetate was 0.565 µmoles at final GC-BET, which is very low as compared to TCKs. Thus the molar concentrations of DMSA-III and DMSA-V were 2.74 µmoles at final GC-BET assay which was almost 5 times the molar concentration of DOATATATE acetate. While molar concentrations for EC and DTPA were as high as 3.73 and 29.51 µmoles at final GC-BET. 

 Highly viscous [^131^I]I-Lipiodol (EL <17 EU/mL) (6, 18) forms two different phases (viscous lipophilic and aqueous hydrophilic phase) on diluting with LRW (MVD - 400), thereby making this product unfeasible to perform GC-BET. We used orbital vortexing at 3000 rpm for 5-8 minutes for [^131^I]I-Lipiodol at first dilution (200) which resulted in uniform dispersion of [^131^I]I-Lipiodol in LRW allowing successful GC-BET of this product. Use of heating prior to performing GC-BET for RFA is not recommended as heating of diluted [^131^I]I-Lipiodol at 125^o^C though may solubilize highly viscous lipiodol but will volatilize highly radioactive Iodine-131 which is very hazardous. Centrifugation of ^131^I-Lipiodol is strictly not recommended for dispersion of RFA as it will lead to separation of hydrophilic and lipophilic phase. BET by KCM using PTS cannot be performed for [^131^I]I-Lipiodol at 400 MVD, since these highly viscous RFA will not mix thoroughly with lyophilized LAL, synthetic CS and CSE present in channel of PTS test cartridges (PTS-TC) and lead to blockage of channel which has diameter of 2 mm.

## Conclusion

 The present study is the first one to report that simple 3000 rpm orbital vortexing can solve the problem of performing GC-BET for [^131^I]I-Lipiodol. Further, this is also the first study to report addition of 2 mM CaCl_2_ solution for [^177^Lu]Lu-DOTATATE to overcome the enhanced false positive result due to increased transglutaminase enzymatic activity and formation of 4 to 6 fold stiffer gel. The adaptation of these modified LAL test methods provides a reliable BET quality control testing option for both the RPs at Hospital Radiopharmacy Settings. Thus an expensive assay like KCM-BET can be replaced by these modified GC-BET for the RPs mentioned in our study.

## Conflict of Interest

 No potential conflicts of interest were disclosed.
